# Supplementary material for: MedTric : A clinically applicable metric for evaluation of multi-label computational diagnostic systems
Source: PLoS One. 2023 Aug 10;18(8):e0283895. doi: 10.1371/journal.pone.0283895 (PMC10414580; doi:10.1371/journal.pone.0283895)
Supplement: S1 Appendix — This file contains basic definitions and terminology associated with binary classification. (PDF) [file pone.0283895.s001.pdf]

Supporting Information

S1 Appendix. Binary Classification

In a binary classification scenario, where a condition can be present (positive) or absent (negative) two metrics are of utmost importance, specificity and sensitivity. Sensitivity

S1 Table. Binary classification categories

Predicted/Ground Truth	True	False
Positive	True Positive (tp)	False Positive (fp)
Negative	False Negative (fn)	True Negative (tn)

gives us the probability of a sick person getting a positive diagnosis, whereas specificity gives us the probability of a healthy person getting a negative diagnostic result.

$$\text{Sensitivity} = \frac{tp}{tp + fn} \tag{1}$$

$$\text{Specificity} = \frac{tn}{tn + fp} \tag{2}$$

Specificity is heavily biased by prevalence, as the number of true negatives is far greater than the number of true positives in a diagnostic context. It is therefore recommended to use precision.

$$\text{Precision} = \frac{tp}{tp + fp} \tag{3}$$

These serve as the basis for several multi-label metrics defined in the paper.
