# Supplementary material for: MedTric : A clinically applicable metric for evaluation of multi-label computational diagnostic systems
Source: PLoS One. 2023 Aug 10;18(8):e0283895. doi: 10.1371/journal.pone.0283895 (PMC10414580; doi:10.1371/journal.pone.0283895)
Supplement: S2 Appendix — This file contains details of parameters used in our experiments, like values for wij, Cij, etc. (PDF) [file pone.0283895.s002.pdf]

S2 Appendix. Dataset Descriptions and Implementation Details.

The PhysioNet 2020/21 data [1] consists of 12 lead ECG signals pooled from several publicly available datasets. Each one of them are marked with potentially several diagnoses from a set of 27 potential diagnoses (see S2 Table).

S2 Table. Possible diagnostic conditions in the PhysioNet dataset.

Diagnosis	Abbr.	Diagnosis	Abbr.
1st Degree AV block	IAVB	Atrial fibrillation	AF
Atrial flutter	AFL	Bradycardia	Brady
Complete right bundle branch block	CRBBB	Incomplete right bundle branch block	IRBBB
Left anterior fascicular block	LAnFB	Left axis deviation	LAD
Left bundle branch block	LBBB	Low QRS voltage	LQRSV
Non specific intraventricular conduction disorder	NSIVCB	Pacing rhythm	PR
Premature atrial contraction	PAC	Premature ventricular contractions	PVC
Prolonged PR interval	LPR	Prolonged QT interval	LQT
Q wave abnormal	QAb	Right axis deviation	RAD
Right bundle branch block	RBBB	Sinus arrhythmia	SA
Sinus bradycardia	SB	Sinus rhythm	NSR
Sinus Tachycardia	STach	Supra-ventricular premature beats	SVPB
T wave abnormal	TAb	T wave inversion	TInv
Ventricular premature beats	VPB		

The weight matrix used for computation of CM and M_{med} is borrowed from the work by Alday et al. [1] The contradiction matrix C_{ij} was constructed with inputs from experts in the field and equals 1 when the pair a_i, a_j cannot occur simultaneously (see S3 Table).

S3 Table. Contradictory pairs in PhysioNet dataset

(AF , IAVB)	(PR , AF)	(PR , AFL)	(PR , CRBBB)
(PR , IRBBB)	(PR , LAnFB)	(PAC , AF)	(PAC , AFL)
(LPR , AF)	(LPR , AFL)	(SA , AF)	(SA , AFL)
(SA , PR)	(SB , AF)	(SB , AFL)	(SB , PR)
(NSR , IAVB)	(NSR , AF)	(NSR , AFL)	(NSR , Brady)
(NSR , CRBBB)	(NSR , IRBBB)	(NSR , LAnFB)	(NSR , LAD)
(NSR , NSIVCB)	(NSR , PAC)	(NSR , PVC)	(NSR , LPR)
(NSR , LQT)	(NSR , QAb)	(NSR , RAD)	(NSR , SA)
(STach , AF)	(STach , AFL)	(STach , Brady)	(STach , PR)
(STach , SB)	(STach , NSR)	(TAb , NSR)	(TInv , NSR)
(NSR , LBBB)	(NSR , SB)		

The significance values were determined by breaking down the possible diagnoses into three groups, namely - super critical, critical and non critical. A weight of 1 was assigned to super critical, 0.8 to critical and 0.6 for non critical conditions. Their constituents are given in S4 Table

The CheXpert [2] dataset contains 224,316 chest radiographs of 65,240 patients. They have uncertainty labels, along with positive and negative labels for all 14 classes.

S4 Table. Significance weights for different diagnoses - 1 is assigned to super critical group, 0.8 to critical group and 0.6 to non critical group.

Super Critical	Critical	Non critical
LQRSV	SA	STach
TAb	Brady	PR
AF	LQT	PVC
AFL	IAVB	SVPB
LBBB	SB	LPR
CRBBB	QAb	VPB
	LAnFB	RAD
		NSIVCB
		IRBBB
		PAC
		RBBB
		LAD
		NSR

We assume all uncertain labels to be false (zeros strategy as described in the paper). The NLP dataset [3] has 978 instances of clinical free text labeled with 45 ICD-9 codes. For both these datasets, we used $s_i = 1 \, \forall i$, $w_{ij} = \frac{2}{3} \, \forall i, j, i \neq j$ and $w_{ii} = 1 \, \forall i$ C_{ij} was taken to be 0.

References

1. Alday EAP, Gu A, Shah AJ, Robichaux C, Wong AKI, Liu C, et al. Classification of 12-lead ECGs: the PhysioNet/Computing in Cardiology Challenge 2020. Physiological Measurement. 2021;41(12):124003. doi:10.1088/1361-6579/abc960.

2. Irvin JA, Rajpurkar P, Ko M, Yu Y, Ciurea-Ilcus S, Chute C, et al. CheXpert: A Large Chest Radiograph Dataset with Uncertainty Labels and Expert Comparison. ArXiv. 2019;abs/1901.07031.

3. Pestian JP, Brew C, Matykiewicz P, Hovermale D, Johnson N, Cohen KB, et al. A shared task involving multi-label classification of clinical free text. In: Biological, translational, and clinical language processing. Prague, Czech Republic: Association for Computational Linguistics; 2007. p. 97–104. Available from: `https://aclanthology.org/W07-1013`.
