# Supplementary material for: MedTric : A clinically applicable metric for evaluation of multi-label computational diagnostic systems
Source: PLoS One. 2023 Aug 10;18(8):e0283895. doi: 10.1371/journal.pone.0283895 (PMC10414580; doi:10.1371/journal.pone.0283895)
Supplement: S3 Appendix — This file contains tables summarizing the experimental data presented in the paper. (PDF) [file pone.0283895.s003.pdf]

S3 Appendix. Summary of data

S5 Table. Data for τ.

(p, q)	Metric	PhysioNet	CheXpert	Medical NLP
(0.8,0.9)	Hamming Loss	0.0 ± 0.0	0.0 ± 0.0	0.0 ± 0.0
	Subset Accuracy	0.0 ± 0.0	0.0 ± 0.0	0.0 ± 0.0
	CM	0.558 ± 0.051		
	Micro F1	0.602 ± 0.043	0.224 ± 0.016	0.830 ± 0.034
	Macro F1	0.602 ± 0.043	0.224	0.830 ± 0.034
	F1	0.604 ± 0.043	0.236 ± 0.016	0.929 ± 0.021
	Accuracy	0.602 ± 0.043	0.224 ± 0.016	0.830 ± 0.034
	MedTric	1.0 ± 0.0	1.0 ± 0.0	1.0 ± 0.0
(0.8, 0.95)	Hamming Loss	0.0 ± 0.0	0.0 ± 0.0	0.0 ± 0.0
	Subset Accuracy	0.001 ± 0.003	0.0 ± 0.0	0.0 ± 0.0
	CM	0.493 ± 0.036		
	Micro F1	0.585 ± 0.038	0.243 ± 0.059	0.782 ± 0.027
	Macro F1	0.585 ± 0.038	0.235 ± 0.055	0.782 ± 0.027
	F1	0.601 ± 0.035	0.335 ± 0.082	0.784 ± 0.029
	Accuracy	0.585 ± 0.038	0.243 ± 0.059	0.782 ± 0.027
	MedTric	1.0 ± 0.0	1.0 ± 0.0	1.0 ± 0.0
(0.6, 0.9)	Hamming Loss	0.0 ± 0.0	0.0 ± 0.0	0.0 ± 0.0
	Subset Accuracy	0.001 ± 0.003	0.0 ± 0.0	0.0 ± 0.0
	CM	0.313 ± 0.053		
	MicroF1	0.621 ± 0.060	0.228 ± 0.052	0.802 ± 0.034
	Macro F1	0.621 ± 0.060	0.221 ± 0.047	0.802 ± 0.034
	F1	0.621 ± 0.060	0.231 ± 0.054	0.819 ± 0.038
	Accuracy	0.621 ± 0.060	0.228 ± 0.052	0.802 ± 0.034
	MedTric	1.0 ± 0.0	1.0 ± 0.0	1.0 ± 0.0
(0.6, 0.95)	Hamming Loss	0.0 ± 0.0	0.0 ± 0.0	0.0 ± 0.0
	Subset Accuracy	0.0 ± 0.0	0.0 ± 0.0	0.0 ± 0.0
	CM	0.323 ± 0.032	0.0 ± 0.0	0.0 ± 0.0
	MicroF1	0.598 ± 0.037	0.336 ± 0.053	0.778 ± 0.035
	Macro F1	0.598 ± 0.037	0.231 ± 0.059	0.778 ± 0.035
	F1	0.598 ± 0.037	0.338 ± 0.052	0.778 ± 0.035
	Accuracy	0.598 ± 0.037	0.336 ± 0.053	0.778 ± 0.035
	MedTric	1.0 ± 0.0	1.0 ± 0.0	1.0 ± 0.0

S6 Table. Data for dispersion.

p	Metric	PhysioNet	CheXpert	Medical NLP
0.99	Hamming Loss	0.121 ± 0.009	0.122 ± 0.006	0.124 ± 0.006
	Subset Accuracy	0.120 ± 0.009	0.121 ± 0.006	0.123 ± 0.006
	CM	0.138 ± 0.014		
	MicroF1	0.086 ± 0.007	0.086 ± 0.005	0.088 ± 0.004
	Macro F1	0.177 ± 0.009	0.175 ± 0.007	0.184 ± 0.008
	F1	0.120 ± 0.009	0.121 ± 0.006	0.123 ± 0.006
	Accuracy	0.120 ± 0.009	0.121 ± 0.006	0.123 ± 0.006
	MedTric	0.073 ± 0.010	0.069 ± 0.010	0.067 ± 0.003
0.95	Hamming Loss	0.123 ± 0.006	0.122 ± 0.006	0.123 ± 0.007
	Subset Accuracy	0.116 ± 0.006	0.117 ± 0.005	0.117 ± 0.006
	CM	0.140 ± 0.009		
	MicroF1	0.085 ± 0.005	0.085 ± 0.004	0.086 ± 0.005
	Macro F1	0.101 ± 0.013	0.110 ± 0.016	0.105 ± 0.009
	F1	0.119 ± 0.005	0.119 ± 0.006	0.120 ± 0.006
	Accuracy	0.118 ± 0.005	0.118 ± 0.005	0.119 ± 0.006
	MedTric	0.070 ± 0.008	0.068 ± 0.007	0.070 ± 0.010
