# Supplementary material for: MedTric : A clinically applicable metric for evaluation of multi-label computational diagnostic systems
Source: PLoS One. 2023 Aug 10;18(8):e0283895. doi: 10.1371/journal.pone.0283895 (PMC10414580; doi:10.1371/journal.pone.0283895)
Supplement: Attachment — Submitted filename: Response to reviewers.pdf [file pone.0283895.s004.pdf]

Dear Dr. Mingxia Liu,

Thank you for giving us the opportunity to submit a revised draft of our manuscript titled *“MedTric: A clinically applicable metric for multi-label computational diagnostic systems”* to *PLOS One*. We are deeply grateful for the time and effort you and the reviewers have dedicated to providing your valuable feedback on our manuscript. We have been able to incorporate changes to reflect most of the suggestions provided by the reviewers, and we believe our manuscript has been greatly improved thanks to their insightful comments. We have highlighted the changes within the manuscript and we will be referring to the document in order to provide a point-by-point response to the reviewers’ comments and concerns.

<u>Comments from academic editor</u>

Comment 1: *insufficient evaluation with only one dataset.*
Response: We wholeheartedly agree with the assessment, and therefore we have added two more datasets for evaluation from different diagnostic disciplines and modalities. In addition to 12 lead ECG data from PhysioNet CINC 2020/21 (line 354 of the marked up manuscript) we have used data from CheXpert, a large scale chest radiography dataset (line 356) and a multi-label clinical free text classification dataset (line 358). In addition to this we have improved the evaluation of the different metrics in question with new statistical measures (line 372, 388) that illustrates the strengths and weaknesses of the metrics better, and our conclusions have been bolstered with more data (line 395, 403).

Comment 2: *missing discussion of other important metrics such as AUC.*
Response: Thank you for this suggestion. We agree that inclusion of an AUC based discussion would have added value but this is slightly out of scope due to the following reason. AUC provides an aggregate measure of performance across all possible classification thresholds, and indicates how well calibrated the probability outputs are. Computation of AUC (both ROC and PR) hinges on the fact that the diagnostic system in question outputs a probability associated with each class, which may not be true in general. The inference algorithm may be more complicated and choose a set of diagnoses without treating each class as an independent binary classification task, thus making an AUC computation, disconnected from actual predictions. However, if we consider the inference algorithm as part of the blackbox model, we can measure and contrast different paradigms of computational diagnostic systems on a similar footing, as they all need to produce a diagnosis. We have added this note to the manuscript (line 111,123).

Comment 3: *unclear manuscript organization and low figure resolution.*
Response: Thank you for this comment. We have tried to organize the manuscript better by adding more sections and subsections and clearly outlining the motivation behind each section (line 91,189, 263, 292, 312, 340, 341). We have also postponed additional details to the Supporting Information section. We separated the design of experiments and results into their own sections (line 340, 394). All figures have been recreated in a higher (600 dpi) resolution.

Responses from Reviewers

Comment 1: *Is the manuscript technically sound, and do the data support the conclusions?*
Reviewer #1: Yes
Reviewer #2: Partly
Response: Thank you for your responses. We further bolstered the conclusions with evaluation on two more datasets (line 356, 358 of marked up manuscript) and redesigned the experiments (line 340) to provide more insights.

Comment 2: *Has the statistical analysis been performed appropriately and rigorously?*
Reviewer #1: Yes
Reviewer #2: No
Response: We are extremely grateful for your feedback. We have fortified the statistical analysis in our manuscript by adding two statistical measures to support our conclusions (line 372, 388). We have also repeated the experiment across several datasets multiple times to provide further evidence for our conclusions (line 399, 407, S5 Table, S6 Table).

Comment 3: *Have the authors made all data underlying the findings in their manuscript fully available?*
Reviewer #1: Yes
Reviewer #2: Yes
Response: Thank you for your feedback.

Comment 4: Is the manuscript presented in an intelligible fashion and written in standard English?
Reviewer #1: Yes
Reviewer #2: No
Response: We are grateful for your feedback and we have tried to make changes for the sake of clarity whenever possible, and carefully revised the manuscript (line 4, 23, 72, 80, 82, 84, 88, 134, 160, 191, 279, 315, 395, 447, etc)

Comments from Reviewer 1

This paper focuses on the topic of performance evaluation for multi-label diagnostics. Based on the drawbacks of several widely-used metrics which are borrowed from the machine learning community, the authors propose a new metric, i.e., MedTric, which takes into account several factors that are of clinical importance. Effectiveness is verified through the analysis on examples.

Strengths
Comment 1: *The topic of evaluation metrics for medical diagnosis is very interesting which has been overlooked by many research work. Additionally, it has clinical value.*
Comment 2: *The authors have conducted detailed analysis about the drawbacks of the existing metrics which are directly borrowed from the machine learning community. The proposed metric is based on the characteristics of medical diagnosis system.*

Comment 3: *The authors have used real datasets to test and verify the effectiveness of the proposed new metric.*
Response: Thanks for pointing out that the topic of evaluation metrics for medical diagnosis has been overlooked by many research studies. Lack of such an effort is the central motivation for our research, which was prompted by our need to evaluate models that are more aligned with domain knowledge. We found that existing metrics are inadequate in that regard. We have also added more datasets for evaluation, and statistical measures to buttress our findings.

Weaknesses
Comment 4: *The overall structure of the paper is not quite clear. An "experiment" section is suggested to be highlighted with detailed experimental settings.*
Response: This comment proved instrumental in improving our manuscript, and we are extremely grateful for your insight. We have tried to organize the manuscript better by adding more sections and subsections and clearly outlining the motivation behind each section (line 91,189, 263, 292, 312, 340, 341 of marked up manuscript). We have also postponed additional details to the Supporting Information section. As per your suggestion, we separated the design of experiments into its own section (line 340) and highlighted experimental settings (line 362, 360, 364, 371, 384, 387, 391, 398, 404, etc)

Comment 5: *The resolutions of the figures are relatively low. The authors are suggested to improve the quality of these figures.*
Response: We agree with your assessment and have recreated every image at a higher resolution (600 dpi).

Comment 6: *For the analysis part, the discussion of some important metrics are missing. For example, Area under the ROC Curve (AUC) is an important metric for evaluating machine learning-based diagnosis systems. The authors are suggested to add some discussions about such metrics.*
Response: Thank you for this suggestion, and although we agree with your assessment, in a general discussion of metrics for computational diagnostic systems, this is slightly out of scope. AUC provides an aggregate measure of performance across all possible classification thresholds, and indicates how well calibrated the probability outputs are. Computation of AUC (both ROC and PR) hinges on the fact that the diagnostic system in question outputs a probability associated with each class, which may not be true in general. The AUC metrics are linked to the threshold tuning aspect of machine learning based systems, and is in general not extendable to every kind of model. For example, consider an inference algorithm which chooses a set of diagnoses without treating each class as an independent binary classification task, thus making an AUC computation, disconnected from actual predictions.
However, if we consider the inference algorithm as part of the blackbox model, we can measure and contrast different paradigms of computational diagnostic systems on a similar footing, as they all need to produce a diagnosis. We have added this note to the manuscript (line 111,123).

Comment 7: *To verify the advantage of the proposed metric, more datasets should be used for analysis. Since a general-purpose metric can have extensive impact on related*

research, only using one dataset is not sufficient to verify its utility in real-world medical practice.
Response: We are grateful for your comment, and we have added two more datasets to validate the advantages of the proposed metric, and also highlight issues with existing ones; CheXpert, a large scale chest radiography dataset (line 356) and a multi-label clinical free text classification dataset (line 358). Additionally, we have introduced estimates for quantities like probability of monotonicity violations (line 372) and dispersion with dataset variation (line 388) which quantitatively demonstrates our claims.

Comment 8: *In machine learning community, there are some branches, such as cost-sensitive learning which is able to treat different classification results differently. The authors are suggested to add some introductions and discussions about it.*
Response: Thank you for pointing this out. Our proposed metric can be thought of as an extension of the cost sensitive learning setup to the clinical setting, and includes a cost term for every possible subset of diagnoses. We have added a few paragraphs highlighting how they fit together (line 76, 323).

Comments from Reviewer 2

The authors proposed a new evaluation index in this paper.

Comment 1: *However, since only one public data set is used, the experiment is limited, and clear statistical verification of the experimental results is required.*
Response: We agree with your assessment and are grateful for it. We have added two more datasets for evaluation from different diagnostic disciplines and modalities. In addition to 12 lead ECG data from PhysioNet CINC 2020/21 (line 354 of marked up manuscript) we have used data from CheXpert, a large scale chest radiography dataset (line 356) and a multi-label clinical free text classification dataset (line 358). In addition to this we have improved the evaluation of the different metrics in question with new statistical measures like probability of maintaining monotonicity (line 372) and dispersion with change in class frequencies (line 384) that illustrates the strengths and weaknesses of the metrics better, and our conclusions have been bolstered with more data (line 399, 407, S5 Table, S6 Table).

Comment 2: *Also, based on the graph results in figure 5, the proposed medtric indicator does not appear to be superior to other evaluation indicators.*
Response: Figure 5 was meant to study the variation of the value of a metric as a function of prevalence of a certain class. We outlined that the desirable behavior was minimum variance, or a metric that is as close to constant as possible. Based on our experiments it was clear that MedTric had the least variance, however the graph was a little confusing. Hamming loss stands out from the pack of metrics in the fact that it is the only one with a 1/(number of classes) factor, thus making the scores not in the same scale as all other metrics, however if you account for that fact, it indeed has a large variance. Thus, we have removed figure 5 from the manuscript and devised an experiment to directly estimate the value of the dispersion (line 388) and plotted the same for all metrics and all datasets in question (Fig. 4 in the revised manuscript). Now, it is clear that our proposed metric has the

least dispersion, and therefore is most resilient to artifacts arising from prevalence. Thank you for your insightful comments, we believe the change has added to the clarity of the paper.

Comment 3: *The results in figure 6 seem unnecessary.*
Response: We agree with your assessment that Fig. 6 was unnecessary, and have thus removed it from the manuscript, and just appropriately cited the source.

Comment 4: *Finally, authors need to refine the entire manuscript by correcting it with clear English expressions.*
Response: Thank you for your feedback. We have tried to make the manuscript clearer whenever possible, and carefully revised the manuscript (line line 4, 23, 72, 80, 82, 84, 88, 134, 160, 191, 279, 315, 395, 447, etc). We have also tried to organize the manuscript better by adding more sections and subsections and clearly outlining the motivation behind each section (line 91,189, 263, 292, 312, 340, 341). Additionally, we have postponed additional details to the Supporting Information section and added a section on experimental design (line 340) so the motivation and methods are clearer. I am confident you will find that the revisions have improved clarity significantly.

We would like to thank you and the reviewers again for your valuable insights. We cannot overstate the incredible impact your feedback had on our manuscript, both in terms of refinement in presentation and in content. We have implemented almost every suggestion that we received, and the one we couldn’t was done in the interest of keeping the manuscript applicable to a wide class of diagnostic systems. We hope that you will find the revised manuscript is up to the mark.

Thanks,

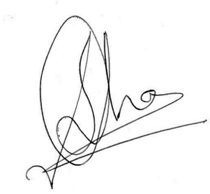

Soumadeep Saha
Corresponding Author
Junior Research Fellow,
Indian Statistical Institute Kolkata
