# Supplementary material for: MedTric : A clinically applicable metric for evaluation of multi-label computational diagnostic systems
Source: PLoS One. 2023 Aug 10;18(8):e0283895. doi: 10.1371/journal.pone.0283895 (PMC10414580; doi:10.1371/journal.pone.0283895)
Supplement: Attachment — Submitted filename: Response_to_reviewers.pdf [file pone.0283895.s006.pdf]

February 21, 2023

Academic Editor
PLOS One

Dear Dr. Mingxia Liu,

Thank you for giving us the opportunity to submit a revised draft of our manuscript titled **“MedTric: A clinically applicable metric for multi-label computational diagnostic systems”** to **PLOS One**. We are deeply grateful for the time and effort you and the reviewers have dedicated to providing your valuable feedback on our manuscript. We have been able to incorporate changes to reflect all of the suggestions provided by the reviewers, and we believe our manuscript has been greatly improved thanks to their insightful comments. We have highlighted the changes within the manuscript and we will be referring to the document in order to provide a point-by-point response to the reviewers' comments and concerns.

Comments from Academic Editor

Several major concerns from the reviewer need to be clarified, such as inconsistent definitions, ...

⧫ We are extremely sorry for this confusion, and are thankful to you for bringing this to our attention. The definition for missed diagnosis and over-diagnosis (line 109-114) in Section 2.1 ($\hat{z}_k \subsetneq y_k$ and $y_k \subsetneq \hat{z}_k$) is the intended definition. The usage in Section 4.1 (line 257) was colloquial, and we used “Missed diagnosis” to mean the diagnoses that were missed (and similarly for over-diagnosis). We have removed this colloquial usage of “missed diagnosis” and “over-diagnosis” (line 257) and have started referring to the sets $y_k - \hat{z}_k, \hat{z}_k - y_k$ as missed predictions and extra predictions to avoid this confusion. For the sake of thoroughness we have added definitions for the same (line 115) and have also amended Figure 2 to reflect this. All our experimental results follow the intended definition, and only one section (Section 4) contained the alternate usage, which has been amended. We hope this will clear up the inconsistency.

... insufficient proof,...

⧫ Thank you for your suggestion and we agree with your assessment. We believe this issue stems from the conflicting usage of missed and over-diagnosis. We have changed the terminology in this revision and our claim has been reworded to state “MedTric always penalizes missed predictions more severely than extra predictions” (line 281). The proof which intended to show that the design of our metric is in line with clinical expectations is now complete (line 284).

Since this alone doesn't demonstrate metric follows required clinical ordering

(line 285), we have also added some discussion on how MedTric maintains clinical severity based ordering using the intended definition (Section 2.1, line 109-114), and showed that within certain conditions MedTric guarantees that this ordering holds, and also pointed out the extremes where it would fail. We hope that this additional analysis will further substantiate our claims. The experimental results we reported consists of a fair head-to-head comparison of all metrics on three datasets, and they clearly show that MedTric always maintains the required order even when no other metric does.

... and confused mathematical expressions. Another round of major revision is expected.

♦ Thank you for your suggestion, we have incorporated all the changes suggested by the reviewers and revised the manuscript to ensure that the mathematical notations are consistent throughout the manuscript. As reviewer #3 pointed out, Equation 22 (line 226) contained a typo, and we have since corrected it. Hamming Loss is symmetric in missed and extra predictions and scores them equivalently, hence the correct equation would have been $|y_i - m_i| = |o_i - y_i|$.

Since the usage of dummy indices were confusing we have reserved the index "i" to refer to dataset instances and the indices "j","k" to refer to diagnostic conditions throughout the paper so further confusion can be alleviated.

We hope that the changes made in this revision add clarity and consistency and the added discussions bolster our claims.

Reviewer's Responses to Questions

1. If the authors have adequately addressed your comments raised in a previous round of review and you feel that this manuscript is now acceptable for publication, you may indicate that here to bypass the "Comments to the Author" section, enter your conflict of interest statement in the "Confidential to Editor" section, and submit your "Accept" recommendation.

Reviewer #1: All comments have been addressed

Reviewer #3: (No Response)

♦ Thank you for your insightful comments in the first round of reviews, your input has been invaluable in improving the manuscript.

2. Is the manuscript technically sound, and do the data support the conclusions?

Reviewer #1: Yes

Reviewer #3: Partly

♦ Thank you for your responses. Hopefully we have been able to address the confusion regarding the definitions, fix the proofs and the mathematical notations. The added discussions should further highlight the strengths of our metric

in a clinical context.

3. Has the statistical analysis been performed appropriately and rigorously?

Reviewer #1: Yes

Reviewer #3: No

♦ Thank you for your responses. We are hopeful that the changes made in this revision that adds clarity to the definitions and analysis will further fortify our conclusions.

4. Have the authors made all data underlying the findings in their manuscript fully available?

Reviewer #1: (No Response)

Reviewer #3: Yes

♦ Thank you for your feedback.

5. Is the manuscript presented in an intelligible fashion and written in standard English?

Reviewer #1: Yes

Reviewer #3: No

♦ We are grateful for your feedback and we have tried to make changes for the sake of clarity whenever possible, and carefully revised the manuscript.

Comments from Reviewer #3

This paper proposed a new metric, i.e., MedTric, to evaluate the multi-label prediction system, which takes the factors that of clinical importance into consideration. Some disadvantages of the general used metrics are discussed. In this revision, most comments from the reviewers have been responded. However, some important issues remain to be addressed.

1. It looks like that the definitions of "Missed Diagnosis" and "Over Diagnosis" are not consistent when they are used to analyze the disadvantages of the existed metrics and when they are used to define the new metric. Specifically, In Section 2.1, Line 111-114, missed diagnosis and over diagnosis are defined as $\hat{z}_k \subsetneq y_k$ and $y_k \subsetneq \hat{z}_k$. However, In Section 4.1, Line 254-255, $y_k - \hat{z}_k, \hat{z}_k - y_k$ correspond to missed diagnosis and over diagnosis, respectively. Also, Fig. 2 is not consistent with the definition in Section 2.1. Since "Missed Diagnosis" and "Over Diagnosis" are the basic concept used in this paper to validate the superiority of the proposed metric, MedTric, this difference should be explained really clear. Otherwise, this inconsistency will bring a big confusion to the readers. Actually, using $y_k - \hat{z}_k$ and $\hat{z}_k - y_k$ to represent missed diagnosis and over diagnosis does seem right me.

♦ We are extremely sorry for this confusion, and are thankful to you for bringing this to our attention. The definition for missed diagnosis and over-diagnosis in Section 2.1 (line 109-114) ($\hat{z}_k \subsetneq y_k$ and $y_k \subsetneq \hat{z}_k$) is the intended definition. The usage in Section 4.1 (line 257) was colloquial, and we used "Missed diagnosis" to mean the diagnoses that were missed (and similarly for over-diagnosis). We have removed this colloquial usage of "missed diagnosis" and "over-diagnosis" and have started referring to the sets $y_k - \hat{z}_k, \hat{z}_k - y_k$ as missed predictions and extra predictions (line 257) to avoid this confusion. For the sake of thoroughness we have added definitions for these terms (line 115) and have amended Figure 2 to reflect this. We hope this will clear up the confusion.

Although $y_k - \hat{z}_k, \hat{z}_k - y_k$ seem intuitive as definitions for missed and over-diagnoses, we are using these terms to talk about the diagnosis as a whole, i.e. $\hat{z}_i$. For example if a patient has conditions $\{A, B, C\}$ and is diagnosed with $\{A, B\}$ we call that a missed diagnosis. This is done for two reasons.

a) It is more obvious what the ordering should be between different diagnoses in a clinical setting by our definition, as by the alternate definition over and missed diagnoses might be present simultaneously. For example if the patient in question receives two diagnoses $\{A, B, D\}$ and $\{A, B\}$ it is a priori hard to say which is of better quality.

b) It is often not feasible to partition the score up so that we can assign each prediction its own score, for example, accuracy is defined as $\frac{1}{N}\sum_i \frac{\hat{z}_i \cap y_i}{\hat{z}_i \cup y_i}$, what is the score due to elements in $\hat{z}_i - y_i$? How do they change if we add an element? To avoid issues like these we felt it was better to talk about the instance as a whole.

2. The proof of the Claim in Section 4.2 is not enough to support the "Claim". Since Equation (39) can only lead to the "Claim" based on the definition of "Missed Diagnosis" and "Over Diagnosis" in Section 4.1 not the one in Section 2.1. Please complete the proof.

♦ We wholeheartedly agree with your assessment and we believe this is due to the conflicting usage of missed and over-diagnosis. We have changed the terminology in this revision and $y_k - \hat{z}_k, \hat{z}_k - y_k$ are now called missed, and extra predictions respectively. Our claim has been reworded to state "MedTric always penalizes missed predictions more severely than extra predictions" (line 281), which was a design goal.

Based on your suggestion we have also added some discussion (line 285-319) on how MedTric maintains clinical severity based ordering using the original definition (Section 2.1, line 109-114), and showed that within certain conditions MedTric guarantees that this ordering holds, and also pointed out the extremes where it would fail. The experimental results we reported consists of a fair head-to-head comparison of all metrics, and they clearly show that MedTric maintains the required order even when no other metric does.

3. There might be some mistake in Equation 22. Since o_i is over diagnosis and $o_i \subsetneq y_i$, it looks like $|y_i - m_i| = |o_i - m_i|$ will not happen. Please make this equation right or explain it.

♦ Thank you for pointing this out, it was a typo, and we apologise for the confusion. Hamming Loss is symmetric in missed and extra predictions and does scores them equivalently, hence the correct equation would have been $|y_i - m_i| = |o_i - y_i|$. We have corrected the same in the manuscript (line 221)

4. In Section 3.2, which is the object that the "inequality" in the sentence "the inequality doesn't hold in general" (Line 225 and 233) represents is not clear to me. Actually, in the example shown in Line 226-227, the inequality (25) holds well. Please make this analysis clearer.

♦ Sorry about the confusion, we were referring to Eq. 6 in that comment.

$$\text{score}_{WD} < \text{score}_{MD} < \text{score}_{OD} < \text{score}_{PD}$$

The example you alluded to do in Line 226-227 and the inequality in Eq. 25 holds well, however this behaviour is contrary to expectations in a clinical setting where we expect missed diagnoses to carry harsher penalties than over-diagnosis. To avoid this confusion further we have named the equation (Equation 6) "Clinical Order" and we have referred to it by name whenever relevant (line 227, 232, 236, etc)

5. The mathematical expressions in this paper needs improvement. There are lots of typos of the mathematical symbols. I'll list some mistakes here, but the modification should not only limit to these.

a) All the x_i in Table 3 should be $\hat{z}_i$.

b) In Line 279-280, z_i should be $\hat{z}_i$

c) g should be g_θ in Eqn. (1).

d) j in Eqn. (23) (26) (28) should be i.

e) Line 244, it's better to replace z_1, z_2 with $\hat{z}_1, \hat{z}_2$.

f) j in Table 3 should be i.

g) The usage of index "i", "j", "k" should be regulated. The misuse led to lots of confusion.

♦ Thank you for your suggestion, and we have made all the changes you suggested and revised the manuscript to ensure that the mathematical notation is consistent across the manuscript. We have also reserved the index "i" to refer to dataset instances and the indices "j","k" to refer to diagnostic conditions throughout the paper so further confusion can be alleviated.

6. The English expression still need to be improved.

♦ Thank you for your input, and we have revised the manuscript again in order

to improve clarity. We hope you will pardon any transgressions arising due to stylistic or subjective choices.

We would like to thank you and the reviewers again for your valuable insights. We cannot overstate the incredible impact your feedback had on our manuscript in terms of presentation refinement, adding clarity, content, and much more. We have been able to incorporate all suggestions we received. We hope that you will find the revised manuscript up to the mark.

Thanks,

Soumadeep Saha
Corresponding Author
Research Fellow
Indian Statistical Institute, Kolkata
