# Supplementary material for: MedTric : A clinically applicable metric for evaluation of multi-label computational diagnostic systems
Source: PLoS One. 2023 Aug 10;18(8):e0283895. doi: 10.1371/journal.pone.0283895 (PMC10414580; doi:10.1371/journal.pone.0283895)
Supplement: Attachment — Submitted filename: Review_comment.docx [file pone.0283895.s005.docx]

This paper proposed a new metric, i.e., MedTric, to evaluate the multi-label prediction system, which takes the factors that of clinical importance into consideration. Some disadvantages of the general used metrics are discussed. In this revision, most comments from the reviewers have been responded.

However, some important issues remain to be addressed.

1. It looks like that the definitions of “Missed Diagnosis” and “Over Diagnosis” are not consistent when they are used to analyze the disadvantages of the existed metrics and when they are used to define the new metric. Specifically, In Section 2.1, Line 111-114, missed diagnosis and over diagnosis are defined as $\hat{z}_{k}⊊y_{k}$ and ${y_{k}⊊\hat{z}}_{k}$. However, In Section 4.1, Line 254-255, ${y_{k}-\hat{z}}_{k}$, $\hat{z}_{k}-y_{k}$ correspond to missed diagnosis and over diagnosis, respectively. Also, Fig. 2 is not consistent with the definition in Section 2.1.

Since Missed Diagnosis” and “Over Diagnosis” are the basic concept used in this paper to validate the superiority of the proposed metric, MedTric, this difference should be explained really clear. Otherwise, this inconsistency will bring a big confusion to the readers. Actually, using ${y_{k}-\hat{z}}_{k}$ and $\hat{z}_{k}-y_{k}$ to represent missed diagnosis and over diagnosis does seem right me.

2. The proof of the Claim in Section 4.2 is not enough to support the “Claim”. Since Equation (39) can only lead to the “Claim” based on the definition of “Missed Diagnosis” and “Over Diagnosis” in Section 4.1 not the one in Section 2.1. Please complete the proof.

3. There might be some mistake in Equation 22. Since $o_{i}$ is over diagnosis and ${o_{i}⊊y}_{i}$, it looks like ${{|y}_{i}-m}_{i}|={{|o}_{i}-m}_{i}|$ will not happen. Please make this equation right or explain it.

4. In Section 3.2, which is the object that the “inequality” in the sentence “the inequality doesn’t hold in general” (Line 225 and 233) represents is not clear to me. Actually, in the example shown in Line 226-227, the inequality (25) holds well. Please make this analysis clearer.

5. The mathematical expressions in this paper needs improvement. There are lots of typos of the mathematical symbols. I’ll list some mistakes here, but the modification should not only limit to these.

1) All the $\hat{x}_{i}$ in Table 3 should be $\hat{z}_{i}$.

2) In Line 279-280, $z_{i}$ should be $\hat{z}_{i}$.

3) $g$ should be $g_{\theta}$ in Eqn. (1).

4) $j$ in Eqn. (23) (26) (28) should be $i$.

5) Line 244, it’s better to replace $z_{1},z_{2}$ with $\hat{z}_{1}$, $\hat{z}_{2}$.

6) $j$ in Table 3 should be $i$.

7) The usage of index “i”, “j”, “k” should be regulated. The misuse led to lots of confusion.

6. The English expression still need to be improved.
